# High-throughput production of human proteins for crystallization: The SGC experience

**DOI:** 10.1016/j.jsb.2010.06.008

**Published:** 2010-10

**Authors:** Pavel Savitsky, James Bray, Christopher D.O. Cooper, Brian D. Marsden, Pravin Mahajan, Nicola A. Burgess-Brown, Opher Gileadi

**Affiliations:** a Structural Genomics Consortium, University of Oxford, Old Road Campus Research Building, Roosevelt Drive, Oxford OX3 7DQ, UK; b Nuffield Department of Clinical Medicine, Old Road Campus, Headington, Oxford OX3 7BN, UK

**Keywords:** Ligation-independent cloning, Structural genomics, Protein expression, Protein purification, Structure prediction, Open source

## Abstract

Producing purified human proteins with high yield and purity remains a considerable challenge. We describe the methods utilized in the Structural Genomics Consortium (SGC) in Oxford, resulting in successful purification of 48% of human proteins attempted; of those, the structures of ∼40% were solved by X-ray crystallography. The main driver has been the parallel processing of multiple (typically 9–20) truncated constructs of each target; modest diversity in vectors and host systems; and standardized purification procedures. We provide method details as well as data on the properties of the constructs leading to crystallized proteins and the impact of methodological variants. These can be used to formulate guidelines for initial approaches to expression of new eukaryotic proteins.

## 1 Introduction

The advent of high-throughput protein crystallography has depended on techniques for systematic expression and purification of proteins at the multi-milligram scale. While purification procedures have traditionally been developed and optimized for each individual protein, the introduction of high-efficiency expression systems and purification tags has allowed a considerable degree of standardization. Comparative summaries of the methods used in many structural genomics groups (Berrow et al., 2006; Graslund et al., 2008) have indicated that a simplified core methodology can be applied to a wide variety of target proteins. The Structural Genomics Consortium (SGC) was set up to solve structures of human proteins with potential therapeutic importance and deposit the structures in public databases without delay (Abbott, 2005; Edwards, 2009; Williamson, 2000). The specific characteristics of this effort have been: (1) A focus on human proteins. (2) Aiming to achieve a success rate of 15–20% of structure determination of proteins from a pre-defined target list. (3) A focus on domains with known or probable biological function. (4) Analysis of multiple members of domain families. (5) Production of purified human proteins for use as reagents in biomedical investigations (Edwards et al., 2009; Fedorov et al., 2007; Uhlen et al., 2008). These goals defined a target list with characteristics that differ from the bulk of targets pursued in other structural genomics efforts, and may require different approaches for protein production. A particular barrier is the difficulty of expressing human proteins in a soluble form in *Escherichia coli*. The SGC operates in three sites, at the Universities of Oxford and Toronto and the Karolinska Institute. Overall the SGC has deposited more than 1000 structures. The SGC group in Oxford has deposited 388 of those, representing 282 distinct protein domains. More than 300 additional protein domains have been expressed and purified but have not yielded crystal structures yet. These proteins provide a rich resource for biochemical investigations, including biophysical characterization, small-molecule inhibitor screens, and generation of antibodies. The target areas investigated at the Oxford group include protein kinases, protein tyrosine phosphatases, small G-proteins, signaling proteins (RGS, SOCS, 14-3-3, PDZ domains), oxidoreductases and other metabolic enzymes, lysine demethylases and DNA helicases. This paper describes the methods used in expression and purification of human proteins in the Oxford group. We focus mainly on bacterial expression and on non-membrane protein domains, with a few comments on expression in the baculovirus vector system.

## 2 Methods

Detailed materials and methods are presented in the Supplementary material. We present here an overview of the methods used in our lab (Gileadi et al., 2008) and discuss some of the rationale and implications of the methods and reagents selected. The procedures used are summarized in Table 1.

### 2.1 Source of cDNA

Human cDNA clones were obtained from various sources. Sixty-eight percent of cDNA clones used in the SGC were derived from the MGC and IMAGE collections (Lennon et al., 1996; Temple et al., 2009); 12% and 6% were obtained from commercial sources and from individual collaborators, respectively; and the remaining 14% were derived from custom synthesis or from RT-PCR of human mRNA. The MGC/IMAGE collection has been an invaluable resource; occasional clones from the collection are missing or mixed up (c. 3%), but all viable clones are sequence-verified and fully documented. Synthetic genes have become more affordable, and offer full flexibility of DNA sequence optimization and specific mutagenesis, as well as access to sequences not represented in available cDNA collections. Although favoured at an early stage, we found that many of the commercial sources are less reliable than the aforementioned sources, and often involve restrictions on use and distribution that are incompatible with our open source policy.

### 2.2 Expression vectors and hosts

Expression clones were generated by PCR and ligation-independent cloning (LIC) into one or more of a set of vectors. The first choice of vector is pNIC28-Bsa4. It is derived from the pET28a vector (Merck), with the expression of the cloned gene driven by the T7-LacO system. Proteins cloned in this vector are fused to an amino-terminal tag of 23 residues (MHHHHHHSSGVDLGTENLYFQ∗SM) including a hexahistidine (His6) and a TEV-protease cleavage site (marked with *). Additional features include cloning sites for ligation-independent cloning (LIC) separated by a “stuffer” fragment that includes the SacB gene. The SacB protein (levansucrase) converts sucrose into a toxic product, allowing selection for recombinant plasmids on agar plates containing 5% sucrose.

Several alternative expression vectors have been used with selected targets (Table 2). pNIC-CTHF appends a C-terminal tag including a TEV-protease cleavage site followed by His6 and a flag epitope. Larger fusion tags include *E. coli* thioredoxin (combined with hexahistidine and a TEV cleavage site), GST, and a reversible streptavidin binding tag (derived from vector pBEN-SBP-SET1, Stratagene). Baculovirus expression vectors were constructed based on pFastBac (invitrogen), incorporating the same arrangement of LIC^2^ cloning sites as the bacterial vectors. We have recently adopted a highly charged, globular domain termed the Z-basic tag (Hedhammar and Hober, 2007), which may provide substantial enrichment of the tagged protein on cation-exchange columns. The Z-basic domain is flanked by a His6 tag and a TEV cleavage site.

An important consideration in vector construction is the ease of cloning the same gene fragment into multiple contexts. LIC requires short (12–16 bp) extensions at both ends of the insert that overlap vector sequences flanking the cloning sites. The vectors used in the SGC can be divided into three LIC classes (Table 2). All vectors within a class utilize the same extensions, so the same PCR fragment can be cloned in parallel into any vector within the class. In practice, cloning a gene into a series of vectors with a variety of N-terminal or C-terminal tags requires at most two PCR reactions (and two pairs of primers). We found this to be nearly as convenient as and more economical than the Gateway system, while minimizing the insertion of extraneous sequences into the expressed proteins.

Host cells are derived from BL21(DE3) and Rosetta2 (Merck). A phage-resistant derivative of BL21(DE3) was isolated in our lab and termed BL21(DE3)-R3; this bacterial strain was then transformed with plasmid pRARE2 (isolated from Rosetta2 calls), which carries seven rare-codon tRNA genes. The resulting chloramphenicol-resistant strain BL21(DE3)-R3-pRARE2 is the standard expression host.

### 2.3 Construct design

One premise of the expression pipeline has been that the protein is the most important factor in determining the success of purification and crystallization. Specifically, it was assumed that cropping a protein into a well-ordered domain may improve the chances of expressing a soluble, stable protein. To implement this principle, each target protein was cloned both as a full-length sequence and as a series of truncated fragments (Graslund et al., 2008). Determining the fragment boundaries was based on bioinformatic prediction of structured domains, primarily on structure-informed sequence alignments with proteins of known structure (GenThreader McGuffin and Jones, 2003), prediction of secondary structures and intrinsically disordered regions (PsiPred, DisorPred, FoldIndex (Jones, 1999; McGuffin et al., 2000; Prilusky et al., 2005), and identification of conserved domains (PFAM, SMART, CD Finn et al., 2008; Schultz et al., 1998; Marchler-Bauer and Bryant, 2004). We define a “fragment” as the segment of the human gene sequence incorporated into an expression clone; a “construct” is the fragment cloned in a cloning vector, which typically appends additional tag sequences on either side. The same fragment may be used in several distinct constructs which may differ in the tags or in the host organism (bacteria of baculovirus). Finally, “domains” are defined as PFAM-annotated structural domains, although the true structural domain may consist of more than one PFAM domain. Three to five alternative boundaries were designated at the predicted boundaries and at nearby positions (2–10 aa apart), and the resulting 9–15 constructs were then cloned and tested for expression and solubility in one or more vectors/hosts. The default cloning vector was pNIC28-Bsa4, which appends an N-terminal hexahistidine tag. Cloning into alternative vectors can be done either in parallel or after obtaining unsatisfactory expression results from the primary vector. The order was normally N-terminal His6, then C-terminal His6, followed by thioredoxin or other large tags. If bacterial expression failed, the gene fragments were cloned into baculovirus vectors.

### 2.4 Cloning and test expression

As in most large-scale structural genomics projects, we have adopted ligation-independent cloning methods to provide directional, in-frame cloning without the specific adjustments required when ligating restriction sites (Graslund et al., 2008; Gileadi et al., 2008; Aslanidis, 1990; Dieckman et al., 2002; Stols et al., 2002). Several groups have adopted the Gateway cloning system (InVitrogen), which is ideally suited for cloning the same fragment into multiple vectors. A disadvantage is the obligatory addition of peptide sequences on one or both ends of the protein, and the use of relatively expensive proprietary reagents for subcloning. We chose a method (Aslanidis, 1990; Stols et al., 2002) based on annealing of short complementary single-stranded 5′-tails generated by the 3′-exonuclease activity of T4 DNA polymerase. The method depends on a 12–16 bp extension added to the PCR primers, which should be composed of three nucleotides only to control the extent of T4 digestion. A similar method (InFusion, Clontech) has no restriction on base composition of the extensions. Having produced more that 40,000 constructs using the T4-based LIC we find that cloning in multiple vectors is easy and efficient, and that the limitations on base composition are insignificant.

Production of many of the human proteins we tested was not straightforward, and in many cases we found that only a small portion of the constructs tested yielded soluble protein. To decide which constructs to use for protein production, and to determine the appropriate cultivation scale, all constructs were evaluated by small-scale expression tests. Optimization of the small-scale experiments is crucial if they are to be reliable predictors of the results of larger-scale production. We largely adopted the 1-ml expression system described in Page et al. (2004). It is important to monitor the growth of the small-scale cultures to ensure that the growth rate and final densities are similar to those obtained in the larger scale (final OD_600_ of 10–12 in TB medium); otherwise, the geometry or agitation speed need to be adjusted to provide adequate aeration. The cell pellets were solubilized with a detergent/nuclease mix (Bugbuster and Benzonaze, Merck Bioscience), centrifuged to remove insoluble material, and the recombinant proteins are purified by Ni-affinity chromatography. The results obtained with this combination were highly correlated with results of subsequent large-scale experiments in which cells (from >1 L cultures) were disrupted mechanically in absence of detergents. In our experience, other detergent formulations tested (e.g. CelLytic from Sigma, or CHAPS) gave much less predictive results, particularly an abundance of “false positive” scores (proteins that cannot be extracted without the specific detergent mix).

### 2.5 Purification

A number of factors are expected to determine the number of steps required to purify a protein to sufficient purity and yield: the protein’s abundance in the initial extract, the availability of affinity purification (usually through an extraneous tag), the degree of binding to contaminating proteins, the stability of the protein at different concentrations and buffer conditions, and the difference in chromatographic properties between the target and contaminating proteins. Expression in an efficient recombinant system, testing multiple constructs to optimize soluble expression, and the use of effective affinity tags (His6) resulted in soluble expression levels of 0.5–50 mg protein/L of culture. At these expression levels (especially at levels greater than 2 mg/L), it has been possible to purify the majority of the proteins that were successfully crystallized using a combination of 2–4 chromatographic steps: nucleic acid removal (by precipitation or anion exchange passage at high salt); Ni-affinity purification, and size-exclusion chromatography. Additional purification could be achieved by cleaving the purification tag with TEV protease, followed by passage on a Ni-affinity resin. As elaborated in the results, some proteins required additional purification, typically ion exchange chromatography.

### 2.6 Quality issues

High-throughput cloning and protein production involves quality issues in every stage. The cDNA clones used as templates for PCR, the synthetic primers, and all of the resulting constructs should ideally be sequence-verified. High-throughput work increases the chances of cross-contamination or pipetting errors. Expressed proteins may be degraded, modified, or accompanied by persistent contaminants. The purified proteins may represent a mixture of aggregation states, conformers, or oxidations states. We have addressed these issues by a combination of DNA sequencing, size-exclusion chromatography, gel electrophoresis, and electrospray-ionization time-of-flight (ESI-TOF) mass spectrometry.

### 2.7 Data management

**Fig. 1:**
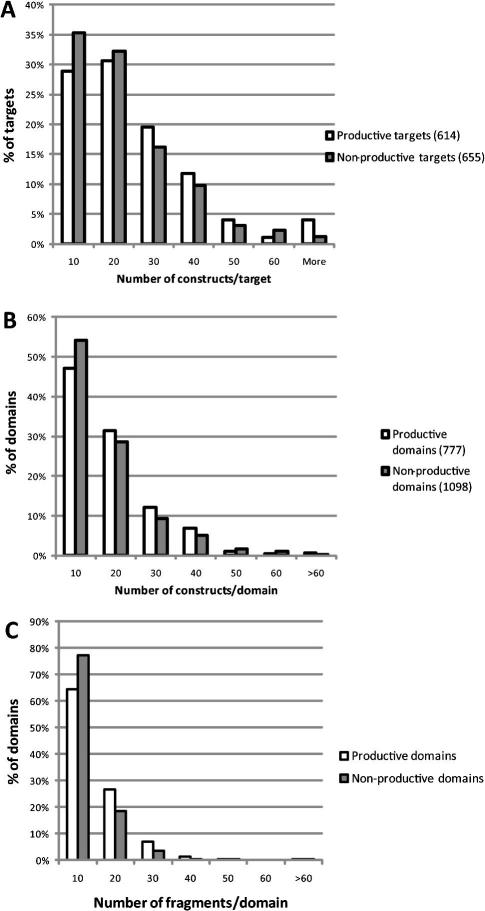
Cloning pipeline. Distribution of the number of constructs generated per each target (A) or each target domain combination (B). Productive targets/domains are those for which at least one construct led to soluble, purified protein; non-productive targets/domains could not be produced in substantial amounts from any of the constructs generated. (C) Distribution of cloned fragments generated per target/domain (same as panel B, but fragments cloned in different vectors are counted only once).

The SGC is a ‘paperless’ environment. Data management is centered upon an Oracle-based database with a data entry and mining tool called BeeHive, which has been extensively modified from a commercial version (http://www.molsoft.com/beehive.html). BeeHive captures all data on targets, including cloning, purification, crystallization and data processing. This enables immediate assembly of all information related to a specific entity (e.g. a clone, protein batch or crystal), as well as complex data mining on the entire database (e.g. the summaries in Figs. 1, 3 and 5). A separate electronic lab notebook (ELN), (http://www.contur.com/) is used to record unstructured data such as experimental details, gel images and chromatograms.

### 2.8 Data and clone dissemination

The full materials and methods and construct information for all structures deposited by the SGC are available on line http://www.sgc.ox.ac.uk/WebGallery/ (Oxford) or http://www.thesgc.org/structures/ (entire SGC). Expression clones for these are available from the SGC or from partner distributors (www.addgene.org) as indicated in the web page or at the email address, contact@sgc.ox.ac.uk. The SGC intends to make available the clones and purification protocols for additional proteins that have not yielded crystal structures.

## 3 Results and discussion

We present some statistics that summarize the degree of effort applied to the protein targets, and some metrics of success. In particular, we attempt to evaluate the impact of several aspects of the high-throughput methods on overall success in protein production and structure determination.

### 3.1 Overall statistics

**Supplementary Figure 1:**
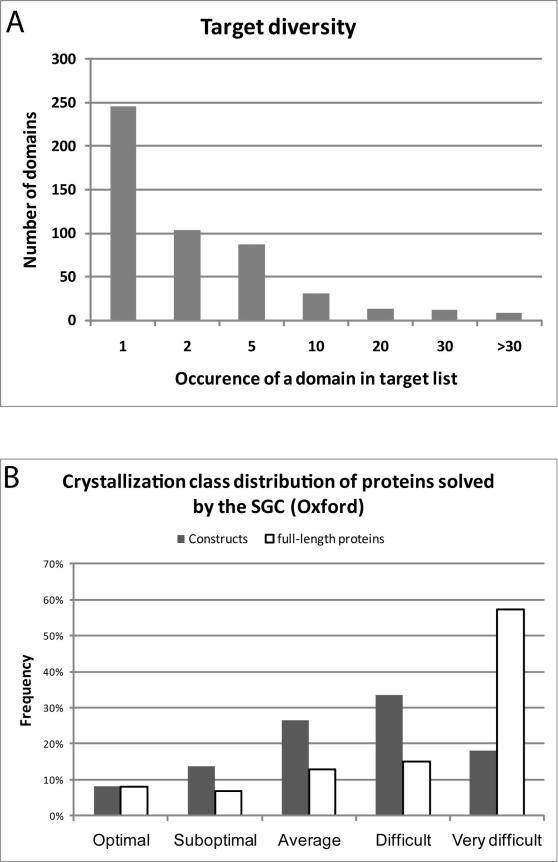
(A) Target diversity; (B) Crystallization class distribution of proteins solved by the SCG (Oxford).

The target list included 1269 distinct human proteins (“targets”), chosen from a variety of families of intracellular proteins (when the target includes a transmembrane component, only the intracellular domains of the target were included). The primary goals of the SGC have been to promote biomedical research by determining the structures of human proteins of medical relevance. Part of this effort was directed at achieving extensive coverage of protein families, aiming to uncover the basis of specificity in biological function and in drug action. However, the target list included, in addition, a wide variety of structural domains: approximately 500 distinct PFAM domains. The balance between family coverage and structural diversity is reflected in the fact that nearly 50% of the domains we examined appear only in one target, whereas nine domains are present in >30 SGC targets, including the catalytic domains of protein kinases, protein tyrosine phosphatases, and short-chain dehydrogenases/reductases, as well as PDZ domains and bromodomains (see more detail in Supplementary Fig. S1, Supplementary Fig. S1, panel A the target list is the balance between proteins with different propensities to crystallize. We have addressed this using XtalPred (Slabinski et al., 2007), looking at all crystal structures solved by the SGC (Supplementary Fig. S1, panel B). The analysis was carried out for the sequences of the crystallized proteins (i.e. the fragment of the protein that was purified and crystallized), as well as the corresponding full-length proteins. One obvious feature is that a large proportion of full-length targets are classified as very difficult, whereas the truncated proteins that were successfully crystallized present a more balanced distribution. This observation probably reflects the fact the protein truncations were designed based on principles similar to those used in evaluating crystallizability.

Expression and purification of each target protein was attempted as multiple truncated constructs in addition to the full-length protein; we define a successful production (“productive target”) when at least one construct of a target gene leads to protein of sufficient quantity and purity to be used in crystallization experiments. 614 of the targets were productive by this measure, a success rate of 48%. The final goal of the project is the determination of the 3D structure of the target proteins; we have deposited 282 non-redundant structures in the PDB, representing 46% of productive targets or an overall success rate of 22%. All but three of the structures were obtained by X-ray crystallography and the remainder by NMR. The full list of non-redundant structures appears in the Supplementary material, Table S3. Note that some structures represent separate domains from one target protein (19 additional separate domains from 9 targets). Success rates calculated considering multidomain proteins can vary between 16% and 22%, depending on the criteria used to count separate domain combinations.

### 3.2 Construct design

How important is the parallel cloning and processing of multiple constructs from each target? The multi-construct approach has been described by Graslund et al. (2008); the present study extends the analysis to a larger set of target proteins. Only 31 (11%) of the 282 structures were obtained by crystallizing the full-length target proteins. An additional 17 structures (6%) were nearly full-length, lacking at most 9 amino acids. Thus, more than 80% of the structures were derived from truncated proteins. The full-length sequence was attempted in most cases (excluding transmembrane and extracellular domains), but often failed either to express or to crystallize. Thus, the generation of truncated proteins is an essential contributor to success.

**Fig. 2:**
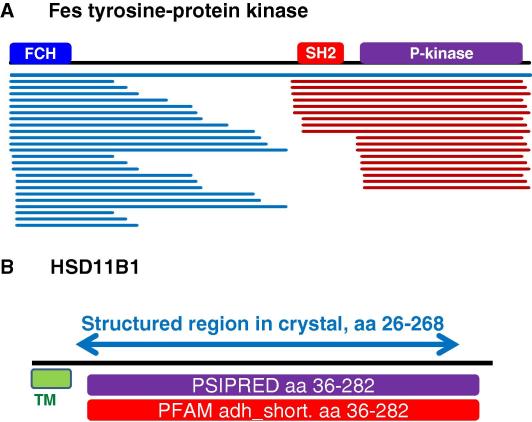
Schematic domain organization and construct design. Fes tyrosine-protein kinase: the black line represents the full-length gene. The three PFAM-annotated domains (FCH, SH2, and P-kinase) are depicted in boxes; the blue and red lines represent the gene fragments that were all cloned and tested for expression and crystallization. Note that the blue and red lines represent distinct constructs. HSD11B1 (11-beta-hydroxysteroid dehydrogenase 1): a demonstration of different definitions of structured domains.

What is a reasonable number of constructs for a target? How should construct boundaries be determined? We first look at statistics of protein expression. Fig. 1A shows the distribution of the number of constructs generated per target; the targets are split into productive and non-productive targets (i.e. targets that yielded purified proteins or not). The numbers of constructs that were made for productive targets tend to be somewhat higher than those for non-productive targets (average constructs/target 22 and 18, respectively). The distributions trail into higher numbers, with significant numbers of targets subcloned as >40 constructs. However, this distribution should be corrected to reflect the multidomain arrangement of many of the target proteins. In addition to the cloning of separate domains in non-overlapping constructs (a minority), there are many cases where different combinations of domains have been tested. For example, the protein kinase Fes (PDB: 3BKB, Ref. Filippakopoulos et al. (2008)) contains three PFAM-listed domains: an N-terminal FCH domain, an SH2 domain, and a C-terminal kinase domain (Fig. 2A). Constructs were made spanning all three domains, all pairwise combinations, and the FCH and kinase domains alone. Thus, although 46 constructs were made of the target Fes, this actually represents 4–5 separate protein entities, each addressed by 10 constructs on average. To correct the target statistics, each target and construct were analyzed for the presence of PFAM domains, and constructs spanning different domain combinations were scored separately (Fig. 1B). Here the number of constructs is mostly below 40 per domain, with an average of 13.5. In most cases, construct numbers larger than 15–20 represent parallel cloning of the same gene fragments into different vectors (Fig. 1C). Fig. 2A also shows that construct termini are closely spaced near well-characterized domain boundaries (e.g. the C-terminus of the kinase domain), but more widely spaced in regions of uncertain organization (between the FCH and SH2 domains).

**Fig. 3:**
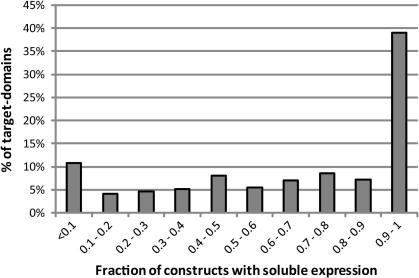
Fraction of soluble constructs per target/domain. For each productive target/domain (as defined in Fig. 1), the fraction of constructs that tested positive for soluble expression was scored. The proportion of domains with different fractions of soluble constructs is displayed in histogram form.

All constructs were tested in small-scale expression experiments (1 ml or 50 ml), and soluble expression was detected in coomassie-stained gels following Ni-affinity purification of the recombinant protein. For each target/domain combination, we have scored the fraction of constructs that expressed detectable levels of soluble protein (a score of 1 means all constructs were positive, a score of 0.1 could mean 1 out of 10 constructs or 4 out of 40, etc.). The distribution of these scores is depicted as a histogram in Fig. 3. We excluded from the analysis all the targets for which no soluble expression could be seen for any of the constructs.

At first sight it is clear that, for a large proportion of productive target/domains (39%), all constructs expressed some level of soluble protein (expression levels may vary). On the other hand, there are ∼15% of target/domains for which less than one in five constructs expressed soluble protein. This analysis seems to indicate that, in absence of prior information, it is reasonable to start a target expression project with 10–20 constructs per domain combination. It is not discussed in this paper, but there are clear advantages in presenting several truncated versions of a protein to crystallization in parallel (Graslund et al., 2008). Consequently, if only 1–2 constructs generate soluble protein, it may be advisable to generate additional constructs with more closely spaced boundaries.

Which construct boundaries work best? We examine here the construct boundaries of the 279 unique deposited crystal structures, asking the following questions. First, how do the construct boundaries relate to the boundaries of known PFAM-annotated domains? Second, how do the constructs boundaries relate to the ends of predicted secondary structure elements? Third, how do the construct ends relate to the actual ends of the structured regions in the crystal structure? And, fourthly, are there any preferred sequence features in the unstructured ends of crystallized constructs? To illustrate the analysis, we can look at the case of HSD11B1 (11-beta-hydroxysteroid dehydrogenase 1), shown schematically in Fig. 2B. The length of the protein from the Genbank entry (gi:5031765) is 292 aa; both predictions and experiments show the presence of a N-terminal transmembrane segment (up to aa 25) that anchors the protein to the ER. The first predicted secondary structure element is a β-strand starting at aa 36; at the C-terminus, a predicted α-helix ends at 282; we refer to the region 36–282 as the predicted structured domain. This region precisely coincides with the region defined by homology to the PFAM domain (pfam00106; adh_short).

The construct used to solve the structure extends from aa 26 to 284. In the crystal structure, (PDB ID: 2BEL) only residues from 26 to 268 are well-ordered; we refer to this region as the “ordered domain”. The differences between the predicted structured domain and the ordered domain result from an additional ordered segment (10 aa) at the N-terminus including both a helical segment and 3 aa with no secondary structure. At the C-terminus, a predicted helix 260–282 is not seen beyond aa 268. These discrepancies are quite common, and reflect a combination of inaccuracies in predictive methods, the presence of structured segments lacking a defined secondary structure, and limitations of crystal structure determination (regions that are not visible may still be structured; additionally, crystal packing may influence structure).

**Fig. 4:**
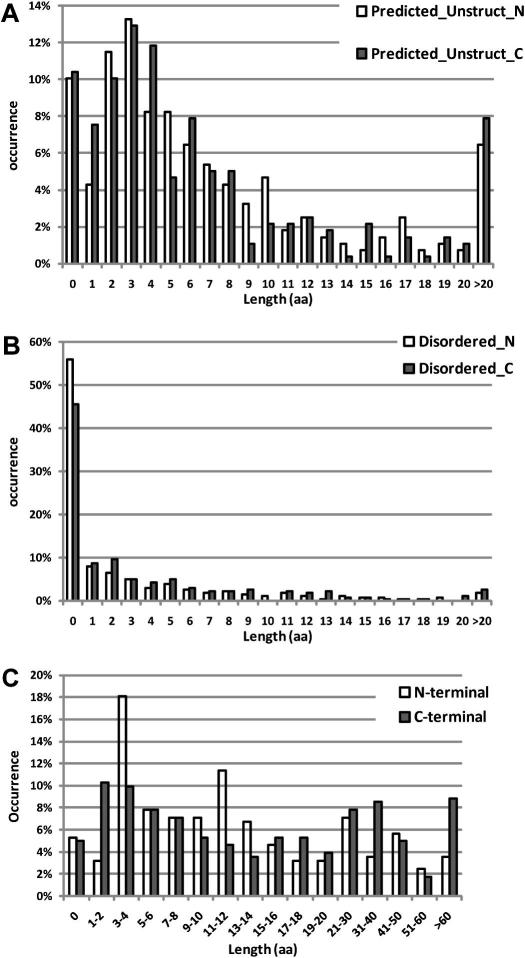
Ends of successful constructs relative to the predicted and observed structured regions. The constructs leading to 282 unique structures were compared with structured regions predicted by PSIPRED (A), the observed structured region in the crystal structure (B), and the annotated PFAM domains (C). The length of extensions in the N-termini (white) and C-termini (full) are depicted as histograms. Note the different scale in panel C.

**Fig. 5:**
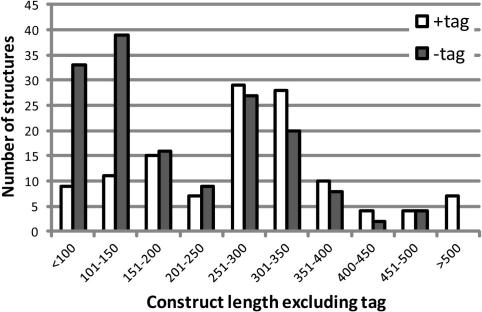
Presence of tags on crystallized proteins. Size distribution of proteins that were crystallized with the tag (open) or following tag cleavage (full).

Fig. 4 presents the distribution of lengths of unstructured termini in the constructs leading to the 279 deposited structures (excluding the tags). Fig. 4A shows the predicted terminal unstructured sequences (these would correspond to aa 26–35 and 283–284 in HSD11B1), while Fig. 4B shows the terminal sequences that are disordered in the crystal structures (corresponding to aa 268–284 in HSD11B1). Fig. 4B demonstrates the generally good coincidence of the boundaries of successful constructs with the boundaries of the ordered regions seen in the crystals: nearly one half of the constructs have no disordered target residues, and 80% have less than six disordered residues on either end. It is not clear whether the short disordered temini occur in the full-length proteins or are a result of the truncation. Fig. 4A shows a wider distribution of distances between the construct termini and the edges of predicted secondary structure; this reflects the discrepancies between secondary structure predictions and observed ordered regions discussed earlier. Fig. 4C shows the distribution of lengths of construct sequences preceding or following the start and end of annotated PFAM domains. This distribution is even wider that that derived from secondary structure predictions, indicating that the domain annotation should always be supplemented with secondary structure predictions when designing constructs. Finally, we looked at the amino acid sequences of the disordered termini. Table 3 presents the amino acid composition of all the disordered termini compared with the overall amino acid composition of the full construct sequences (the full sequences are presented in the Supplementary material, Table S3). A significant trend emerges: the disordered termini have an increased proportion of serine and proline; a significant under-representation of hydrophobic residues (particularly aromatic residues); and only modest changes in the proportion of charged residues. This amino acid distribution is similar to that reported in Linding et al. (2003) for coiled regions, and is used in algorithms that predict disordered regions in proteins.

Although this survey is far from exhaustive, the results can provide some validated guidelines on the design of constructs for protein expression and crystallization.

(1) The crystal structure, if known, provides the best guide to the design of expression constructs. The structure is rarely known in advance, but guidance from alignments with structures of related proteins or from NMR structures can often be used. It is best to design multiple constructs (with 3–4 boundaries on each side), which either coincide with the boundaries of the known structures, or include additional short sequences (typically up to 10 aa).
(2) Multiple sequence alignments and annotated conserved domains (PFAM, CD) are a rough guide to construct design; when several annotated domains exist (and depending on the domain of interest), design separate series of constructs containing different combinations of domains. After the relevant domain boundaries are identified, use secondary structure prediction methods to design construct boundaries that do not disrupt high-likelihood structural elements. Because of the uncertainty of these predictions, multiple constructs should be designed spanning 1–10 aa or even more beyond the predicted ends.
(3) Constructs termini should be preferably chosen to exclude aromatic and other hydrophobic residues near the end, while favouring serine and proline in short, potentially disordered stretches.

### 3.3 Tags, vectors and hosts

The choice of vectors and tags was dictated by the same considerations that were applied in a large number of research groups; as others have done, we have developed a series of vectors with a number of fused tags, which can in most cases be removed by cleavage with TEV protease (summarized in Table 2 and in Supplementary Table S2). The short, cleavable tags allow us to attempt crystallization of the same protein with or without the tag. This proved to be beneficial, as many proteins have crystallized preferentially in one form or the other. The primary choice (and the first tag tested) is an N-terminal 23-aa peptide containing a His6 sequence and a TEV cleavage site. Maintaining the preference for short tags, the second choice has been a C-terminal His6 tag (either a cleavable, 23-aa tag that also includes a Flag epitope, or a short non-cleavable sequence AHHHHHH). If soluble protein cannot be recovered with short tags, longer tags comprising whole protein domains are used: GST or Trx (*E. coli* thioredoxin). The thioredoxin tag is combined with a His6 sequence to allow purification by Ni-affinity chromatography (Vincentelli et al., 2003). It is anecdotally known that proteins that require a large tag for soluble expression may precipitate when the tag is removed. We have not investigated this thoroughly, but significant numbers of targets have been productively produced with GST or Trx tags and survived tag removal (50 out of 70 targets attempted with GST tags, 24 out of 33 targets attempted with a Trx tag).

The impact of tag exchange has been significant: 9% of our deposited structures were obtained with constructs bearing C-terminal His6 tags. In these cases, either the N-terminally tagged constructs did not produce soluble protein, or failed to crystallize. Constructs with large tags – GST and Trx – provided an additional 3% of the deposited structures. The significant chance of rescuing expression by switching the location of the tag has driven us to perform parallel cloning into two vectors (N-terminal and C-terminal His6 tags) at the initial cloning stage, unless prior experience with related proteins directs otherwise. The next favoured option is a Trx fusion, which has rescued expression of several targets and can frequently be cleaved without loss of protein solubility.

The standard hosts for bacterial expression are derivatives of BL21(DE3). We have tested the influence of the different codon biases in human and *E. coli* genes, and found that a significant improvement in total and soluble expression levels could be achieved by either synthesizing codon-optimized genes or providing tRNAs for rare codons in the host cells (Burgess-Brown et al., 2008). We have used either the strain Rosetta 2 (Merck) or a phage-resistant derivative isolated in our lab.

Specific host strains were created to co-express either accessory proteins or heteromeric complexes. Expression of the chaperone proteins GroEL–GroES has been useful in expression of a handful of proteins; however, the chaperone proteins often co-purify with the target protein and are hard to remove. We have not explored this route extensively. Host strains expressing phosphatases (lambda phosphatase or YopA phosphatase) have been useful for expression of some protein kinases, which would otherwise auto-phosphorylate.

In small-scale experiments we have observed significant variation between individual colonies from the transformation of an expression construct into the host cells. While these differences should be examined and the colonies with best expression selected for further work, this may be impractical in a high-throughput setting. Instead, we routinely combine several colonies from the initial transformation, achieving more reproducible results. Glycerol stocks made from fresh transformants can be kept for many months at −80 °C without loss of activity; ideally, several aliquots are prepared of each stock. It is the authors’ experience that there are isolated cases where expression can only be obtained from a fresh transformant (e.g. yeast TATA-binding protein and Taq polymerase; O.G., unpublished observations); this should be checked if expression results from glycerol stocks are inconsistent.

### 3.4 Expression in baculovirus-infected insect cells

**Fig. 6:**
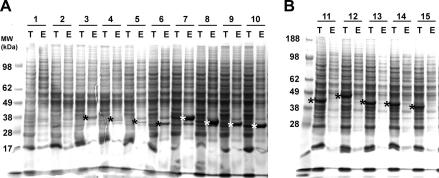
Multiconstruct testing in baculovirus. Ten constructs of one gene (A) and five constructs of a second gene (B) were cloned in baculovirus and expression was tested following infection of 3-ml suspension cultures of SF9 cells (Shrestha et al., 2008). The cells were lysed after 48 h, the lysates were clarified by centrifugation and the recombinant His6-tagged proteins were purified by Ni-affinity. Aliquots of the uncentrifuged total lysate (T) and the NiNTA-eluate (E) were analyzed by SDS–PAGE. The positions of the recombinant proteins are marked with (*).

A significant fraction of human targets could not be expressed in soluble form in *E. coli* using the methods described above (approximately 1/3 of the targets tested did not produce soluble protein in small-scale or large-scale expression tests). We have used, as an alternative method, the baculovirus expression system. A full description of the results is beyond the scope of this paper; the methods for high-throughput cloning and expression testing are described elsewhere (Shrestha et al., 2008). An important observation that has emerged is the contribution of parallel cloning of multiple constructs of each target. Although the statistics are still meagre, the results are similar to our experience with bacterial expression. An example is shown in Fig. 6, where small-scale cultures were used to test several constructs of two targets. Panel A shows the substantial differences in the levels of soluble expression between different constructs: very high levels in constructs 7–11, modest levels in 3–6, and barely detectable or absent in constructs 1–2. Panel B shows that, similar to *E. coli*, protein solubility can be the limiting factor in protein production in insect cells: there is indication of high expression levels in the total extracts, but the protein is lost upon centrifugation and is not seen in the eluted fractions. Thus, effective recovery and crystallization of human proteins from the baculovirus system also requires creation and testing multiple truncated constructs.

### 3.5 Purification

Protein purification may seem to be difficult to standardize, because of the wide variation in the physical and chemical properties of different proteins. In fact, the combination of recombinant expression and effective purification tags allowed us to use a limited set of purification protocols to achieve sufficient purity and yield for protein crystallization (roughly scored as >95% pure, although often of higher purity). Specific considerations when assessing purity of proteins for crystallization are the chemical and biophysical homogeneity of the target protein, rather than the complete absence of other contaminating proteins. Chemical homogeneity here means the absence of multiple chemical species differing by oxidation, proteolysis or post-translational modification (e.g. phosphorylation), as monitored by mass spectrometry of the intact proteins. If heterogeneity is detected, it can be resolved by further chromatographic steps (e.g. ion exchange chromatography to resolve different phosphorylated forms), by enzymatic treatment (e.g. phosphatase), or by modifying the expression and purification procedure (e.g. additional protease inhibitors and reducing agents).

Eighty-nine percent of the proteins leading to crystal structures were purified by variants of two generic procedures. 40% of proteins were purified by a two-column procedure: Ni-affinity purification followed by size-exclusion chromatography. The second procedure includes, in addition, tag cleavage and re-purification. This is a very effective means of removing contaminants that bind non-specifically to the Ni columns. Such procedures were used for 49% of the structures; the exact sequence of columns may vary (see detailed protocols in the Supplementary material). For 9% of the crystallized proteins, these generic procedures were not sufficient to achieve the desired purity; in most of these cases, ion exchange was used as an additional step.

The generic protocols work well for proteins that are at least modestly expressed in bacteria. However, difficulties arise when the expression levels are low, necessitating the processing of cultures larger than 5 L; extraction and purification of tagged proteins from baculovirus-infected insect cells present similar issues. The low abundance of the proteins in the initial extracts often leads to insufficient purity after the first steps. In addition, loading large volumes of cell lysates on Ni-Sepharose resins leads to interference with binding, probably due to stripping the Ni ions by some component of the lysate (Magnusdottir et al., 2009). To alleviate some of these problems, we have adopted the Z-basic purification tag (Hedhammar and Hober, 2007). This 54-aa sequence, derived from Staphylococcal protein A which was engineered to have high positive surface charge, allows tagged proteins to bind to cation-exchange resins at salt concentrations in which most cellular proteins do not. A pair of LIC-vectors for use in *E. coli* and baculovirus incorporate a His10 sequence, the Z-basic tag, and a TEV cleavage site. Preliminary evidence suggest a marked degree of purification by combining cation exchange and Ni-affinity purification steps, especially with proteins extracted from baculovirus expression.

Protein stability and solubility may limit the yield or utility of purified proteins. These are best monitored by following the activity of the protein through purification and storage. However, activity assays may not be available or practical for many of the proteins; in these cases, alternative biophysical methods were used. Indicators of poor stability and solubility are aberrant size-exclusion chromatography profiles, aggregation seen in dynamic light scattering (DLS), concentration- or time-dependent precipitation from solution, loss of protein during purification, and inability to concentrate the protein using ultrafiltration. At this point it becomes essential to find buffer conditions or additives that increase protein stability and solubility. In some cases it has been possible to identify stabilizing conditions by systematic screens using fluorescence or static light scattering (Vedadi et al., 2006). In other cases, a more limited survey of conditions used precipitation or ultrafiltration as readout. In addition to testing pH, salt composition and concentration, we have occasionally seen a positive effect of 25% glycerol, 0.5 M trimethylamine *N*-oxide (TMAO) (Yancey, 2005), or a mixture of arginine and glutamate (0.2 M each); this obviously represents a small subset of potential stabilizing additives. We have generally avoided the use of detergents to avoid potential complications in crystallization of non-membrane proteins.

### 3.6 Quality issues

Various steps in the route from gene to purified protein can introduce errors. Genes and cDNA clones appear in several variants, including splice variants and point differences. When possible, cDNAs were selected or synthesized according to the Reference Sequence (RefSeq, Pruitt et al., 2007). However, variants were tolerated when a cDNA matching the RefSeq is unavailable, and when the variants are documented, or appear in closely related orthologues. All cDNA entry clones were fully sequenced, either by the supplier or in-house.

The cloning process is routinely monitored by the size of PCR-generated DNA gel bands. Combined with the clear pedigree of each construct, known from its position in successive 96-well plates, this indicates the presence of inserts and the lack of gross deletions or insertions. At a finer scale, PCR-based subcloning can introduce sequence errors both within the primer sequences and in the amplified region. Sequencing of a subset of constructs revealed significant error rates: approximately 5% of constructs contained errors in the primer sequences (often 1-base deletions), and a further 5% contained errors within the amplified region (usually missense). The error rate was similar for different suppliers of oligonucleotide primers. It may be possible to reduce the error rates somewhat by further purification of the primers or by using different DNA polymerase formulations for the PCR reactions; we have not tested these possibilities systematically. Ideally, all constructs should be fully sequenced. In practice this has not been possible; instead, all constructs leading to crystallized proteins, and all constructs which produce proteins with aberrant masses, were sequenced. In addition, a random subset of constructs was sequenced to identify major changes in quality of the cloning reagents.

The identity and quality of every protein batch is tested by mass spectrometry. Measurement of intact mass by electrospray-ionization time-of-flight (ESI-TOF) results in single-Dalton accuracy and is a clear indicator of irregularities. It is crucial to attempt to account for any deviation from the predicted molecular weight. Some changes indicate defined post-translational modification (e.g. phosphorylation, removal of initiator methionine, acetylation, and glycosylation), whereas others indicate protein degradation or oxidation. Actions can be taken to reduce the heterogeneity of the protein or to generate a desired form of the protein: for example, dephosphorylation, autophosphorylation, site-directed mutagenesis, and improving protease protection.

Many mass discrepancies cannot be accounted for by defined post-translational modifications. In such cases, it is crucial to sequence the expression plasmid. As indicated earlier, there is a significant incidence of primer- or PCR-derived mutations, including missense and frameshift. Finally, in a high-throughput operation there will be occasional mis-labeling or cross-contamination of clones; these can sometimes be identified directly from the intact mass. Tandem MS of trypsin-digested proteins has been used in a more restricted basis; the main uses include identification of proteins in gel bands from low-level expression and identification and mapping of post-translational modifications.

A crucial quality issue encountered by several laboratories is bacteriophage infection. In a high-throughput environment it is not easy to maintain strict microbiological practices. Bacteriophages can be introduced either from the environment or through clones obtained from other labs; initial sporadic infections (evident as failed bacterial cultures) can easily spread to a lab-wide infestation which is extremely difficult to eliminate. Following a wide-spread infection with a T1-related phage, we have introduced two measures. First, we isolated a spontaneous phage-resistant mutant of BL21(DE3) (reproducing the experiment of (Luria and Delbruck, 1943), and have used this strain as the major expression strain. In addition, all cloning is done in phage T1-resistant host strains. Second, bacterial cultures showing any sign of bacteriophage infection (growth arrest or lysis, slimy or stringy appearance) are decontaminated and aliquots are tested for formation of plaques. These measures, together with increased awareness and better microbiological practice, have helped us avoid subsequent infections for over 5 years. Our strains are, of course, not universally resistant, but a very rapid selection can be applied if new infections occur.

## 4 Concluding remarks

Production of purified human proteins for crystallization and biochemical studies is a challenging prospect. Data presented here and elsewhere (e.g. Graslund et al. (2008), Banci et al. (2006)) show that parallel processing of a modest number of constructs can improve the recovery of soluble targets in both bacterial and baculovirus expression systems. Arguably, the selection of a well-expressed stable version of a protein makes it easier to standardize expression and purification protocols.

Are we approaching a “glass ceiling”, with a considerable proportion of human targets inaccessible to recombinant expression? There are clearly procedural optimizations that were not addressed in our high-throughput work, which may lead to incremental improvements in target expression. Many of these approaches may be applicable in projects involving a small number of target proteins; some can be incorporated in a high-throughput pipeline.

We have set a cut-off for productive targets such that several milligrams of protein can be obtained from 1 to 10 L of bacterial or insect cell culture; however, with fermentation technologies, culture volumes of tens to hundreds of liters can be easily handled, capturing targets expressing at lower levels. In addition, protein expression and purification can be further optimized by utilizing additional tags, host strains, promoters, and culture conditions (Vincentelli et al., 2003).

Construct screening has proved an effective way of selecting well-expressed versions of target proteins; this principle has been extended to more extensive screening of constructs, down to full coverage at single-residue level (Cabantous and Waldo, 2006; Cornvik et al., 2006; Hart and Tarendeau, 2006; Reich et al., 2006). Although these methods have led to some remarkable successes, their impact in large-scale target recovery remains to be assessed. A wide area of protein engineering that has been only modestly exploited is mutagenesis within the protein sequence; site-specific mutations (e.g. replacement of phosphorylation or glycosylation sites, and deletion of disordered or hydrophobic loops) as well as randomized mutagenesis may improve protein recovery.

Another approach that may be valuable is converting the host cells to become more hospitable to expression of particular targets by expressing a wider range of molecular chaperones, including target-specific chaperones (e.g. HSP90 and CDC37 for protein kinases; Ref. Pearl (2005)) or modifying enzymes.

A fundamentally different approach that may dominate future progress is the expression and analysis of protein complexes (e.g. http://www.spine2.eu). Many target proteins never occur in the cells as isolated polypeptides, and co-expression may be the only way to maintain the native fold of such proteins.

Finally, although the criterion of therapeutic value has prescribed a focus on human proteins, there may be cases when orthologues from other organisms may be the only choice; small differences in sequence may significantly affect success in expression and crystallization (Savchenko et al., 2003).

## Figures and Tables

**Table 1:** Summary of experimental approaches.

Sourcing human cDNA clones	1. IMAGE collection; 2. Commercial collections 3. Custom gene synthesis 4. PCR/cloning from cDNA.
Construct design	Designate 2–5 boundaries on either end, based on predictions of domain boundaries, secondary structure prediction, and sequence alignment. Generate 9–15 constructs for each target or domain.
Vectors and hosts	Primarily *E. coli* (BL21 derivatives expressing rare-codon tRNAs); alternative expression in baculovirus-infected insect cells is discussed separately. Vectors (see Table 2 for details).
Cloning	Ligation-independent cloning (LIC), using complementary ssDNA ends generated by T4 DNA polymerase.
Evaluating clones	Presence and size of inserts verified by PCR, and a subset of constructs are sequenced. Soluble protein expression tested in 1-ml cultures or 50-ml cultures. Expression is performed in rich medium (TB) at 18–25 °C. The cells are lysed in mild detergent (BugBuster®) followed by NiNTA purification.
Protein expression (*E. coli*)	1–5 L cultures in TB medium, grown to OD 3 at 37 °C. Induction with IPTG at lower temperatures (18–25 °C) overnight.
Protein purification (*E. coli*)	Cells are extracted by high-pressure or sonication without detergents. Standard purification includes IMAC and gel filtration at high salt (0.3–0.5 M NaCl). Some proteins are further treated with TEV protease to remove the tag, then passed through IMAC again to remove impurities, and gel filtration if necessary. When the standard methods are insufficient, additional steps such as ion exchange, heparin-Sepharose or Blue-Sepharose may be applied.

**Table 2:** Expression vectors.

Name	Tag description	LIC class	Ab resistance and compatibility	Accession Nos.
pNIC28-Bsa4	N-terminal His6, TEV-cleavable (23 aa) MHHHHHHSSGVDLGTENLYFQ∗SM	1	Kanamycin; pET	EF198106
pLIC-SGC1	N-terminal His6, TEV-cleavable (23 aa) MHHHHHHSSGVDLGTENLYFQ∗SM	1	Ampicillin; pET	EF456737
pNIC-CTHF	C-terminal His6-Flag, TEV-cleavable. AENLYFQ∗SHHHHHHDYKDDDDK	2	Kanamycin; pET	EF199844
pNIC-CH	C-terminal His6, not cleavable. HHHHHH	3	Kanamycin; pET	EF199843
pNIC-NH-TrxT	N-terminal His6, Thioredoxin, TEV-cleavable	1	Kanamycin; pET	GU269914
pNIC-Zb	N-terminal His6, Z-basic domain (Hedhammar and Hober, 2007), TEV-cleavable.	1	Kanamycin; pET	GU452710
pBEN1-SGC	N-terminal SET (solubility-enhancing) and SBP (streptavidine-binding peptide) tags, TEV-cleavable.	1	Kanamycin, pET	GU725055
pFB-LIC-Bse	Baculovirus cloning vector. N-terminal His6, TEV-cleavable (22 aa). MGHHHHHHSSGVDLGTENLYFQ∗SM	1	Ampicillin; transposition in the bacterial strain DH10Bac (InVitrogen) to generate recombinant baculovirus DNA	EF199842

**Table 3:** Amino acid composition of unstructured termini (tags excluded).

Amino acid	1	2	3	4	5
N-terminal disordered	C-terminal disordered	Total disordered termini	Entire protein sequences	Enrichment in termini (3/4)
Ser	11.9% (93)	10.8% (110)	11.3% (203)	6.1% (4055)	1.85
Pro	9.6% (75)	6.3% (64)	7.7% (139)	4.6% (3028)	1.70
Gln	6.1% (48)	5.4% (55)	5.7% (103)	4.3% (2854)	1.33
Glu	9.2% (72)	9.4% (95)	9.3% (167)	7.1% (4735)	1.30
Thr	4.8% (38)	7.5% (76)	6.3% (114)	5.0% (3322)	1.27
Lys	7.0% (55)	8.2% (83)	7.7% (138)	6.3% (4200)	1.21
Gly	9.9% (78)	7.9% (80)	8.8% (158)	7.3% (4835)	1.21
Ala	9.7% (76)	6.3% (64)	7.8% (140)	7.3% (4873)	1.06
Asn	3.1% (24)	4.2% (43)	3.7% (67)	3.7% (2466)	1.00
Met	2.6% (20)	2.3% (23)	2.4% (43)	2.4% (1615)	0.98
Arg	4.2% (33)	5.0% (51)	4.7% (84)	5.2% (3450)	0.90
Asp	5.1% (40)	4.5% (46)	4.8% (86)	5.3% (3543)	0.90
His	2.4% (19)	1.9% (19)	2.1% (38)	2.5% (1664)	0.84
Val	3.7% (29)	6.2% (63)	5.1% (92)	7.4% (4913)	0.69
Leu	4.8% (38)	6.5% (66)	5.8% (104)	9.5% (6342)	0.61
Cys	1.0% (8)	1.1% (11)	1.0% (19)	1.8% (1220)	0.58
Phe	1.4% (11)	2.3% (23)	1.9% (34)	3.9% (2574)	0.49
Ile	1.8% (14)	2.6% (26)	2.2% (40)	5.6% (3748)	0.39
Trp	0.38% (3)	0.49% (5)	0.44% (8)	1.2% (795)	0.37
Tyr	1.3% (10)	1.2% (12)	1.2% (22)	3.4% (2247)	0.36

## Appendix A Supplementary data

Supplementary data 1 Vector information sheet.

Supplementary data 2 Experimental details for LIC cloning vectors (see Table 1 in the text for full vector details).

Supplementary data 3 Structures solved by SGC: details of construct sequence and protein purification schemes.

## Footnotes

2 *Abbreviations used:* LIC, ligation-independent cloning; PCR, polymerase chain reaction; TCEP, tris(2-carboxyethyl)phosphine; TEV, tobacco etch virus; aa, amino acid(s).

## References

[bib1] Abbott A. (2005). Protein structures hint at the shape of things to come. Nature.

[bib2] Aslanidis C., de Jong P.J. (1990). Ligation-independent cloning of PCR products (LIC-PCR). Nucleic Acids Res..

[bib3] Banci L., Bertini I., Cusack S., de Jong R.N., Heinemann U., Jones E.Y., Kozielski F., Maskos K., Messerschmidt A., Owens R., Perrakis A., Poterszman A., Schneider G., Siebold C., Silman I., Sixma T., Stewart-Jones G., Sussman J.L., Thierry J.C., Moras D. (2006). First steps towards effective methods in exploiting high-throughput technologies for the determination of human protein structures of high biomedical value. Acta Crystallogr. D Biol. Crystallogr..

[bib4] Berrow N.S., Bussow K., Coutard B., Diprose J., Ekberg M., Folkers G.E., Levy N., Lieu V., Owens R.J., Peleg Y., Pinaglia C., Quevillon-Cheruel S., Salim L., Scheich C., Vincentelli R., Busso D. (2006). Recombinant protein expression and solubility screening in *Escherichia coli*: a comparative study. Acta Crystallogr. D Biol. Crystallogr..

[bib5] Burgess-Brown N.A., Sharma S., Sobott F., Loenarz C., Oppermann U., Gileadi O. (2008). Codon optimization can improve expression of human genes in *Escherichia coli*: a multi-gene study. Protein Expr. Purif..

[bib6] Cabantous S., Waldo G.S. (2006). In vivo and in vitro protein solubility assays using split GFP. Nat. Methods.

[bib7] Cornvik T., Dahlroth S.L., Magnusdottir A., Flodin S., Engvall B., Lieu V., Ekberg M., Nordlund P. (2006). An efficient and generic strategy for producing soluble human proteins and domains in *E. coli* by screening construct libraries. Proteins.

[bib8] Dieckman L., Gu M., Stols L., Donnelly M.I., Collart F.R. (2002). High throughput methods for gene cloning and expression. Protein Expr. Purif..

[bib9] Edwards A. (2009). Large-scale structural biology of the human proteome. Annu. Rev. Biochem..

[bib10] Edwards A.M., Bountra C., Kerr D.J., Willson T.M. (2009). Open access chemical and clinical probes to support drug discovery. Nat. Chem. Biol..

[bib11] Fedorov O., Marsden B., Pogacic V., Rellos P., Muller S., Bullock A.N., Schwaller J., Sundstrom M., Knapp S. (2007). A systematic interaction map of validated kinase inhibitors with Ser/Thr kinases. Proc. Natl. Acad. Sci. USA.

[bib12] Filippakopoulos P., Kofler M., Hantschel O., Gish G.D., Grebien F., Salah E., Neudecker P., Kay L.E., Turk B.E., Superti-Furga G., Pawson T., Knapp S. (2008). Structural coupling of SH2-kinase domains links Fes and Abl substrate recognition and kinase activation. Cell.

[bib13] Finn R.D., Tate J., Mistry J., Coggill P.C., Sammut S.J., Hotz H.R., Ceric G., Forslund K., Eddy S.R., Sonnhammer E.L., Bateman A. (2008). The Pfam protein families database. Nucleic Acids Res..

[bib14] Gileadi O., Burgess-Brown N.A., Colebrook S.M., Berridge G., Savitsky P., Smee C.E., Loppnau P., Johansson C., Salah E., Pantic N.H. (2008). High throughput production of recombinant human proteins for crystallography. Methods Mol. Biol..

[bib15] Graslund S., Sagemark J., Berglund H., Dahlgren L.G., Flores A., Hammarstrom M., Johansson I., Kotenyova T., Nilsson M., Nordlund P., Weigelt J. (2008). The use of systematic N- and C-terminal deletions to promote production and structural studies of recombinant proteins. Protein Expr. Purif..

[bib16] Graslund S., Nordlund P., Weigelt J., Hallberg B.M., Bray J., Gileadi O., Knapp S., Oppermann U., Arrowsmith C., Hui R., Ming J., dhe-Paganon S., Park H.W., Savchenko A., Yee A., Edwards A., Vincentelli R., Cambillau C., Kim R., Kim S.H., Rao Z., Shi Y., Terwilliger T.C., Kim C.Y., Hung L.W., Waldo G.S., Peleg Y., Albeck S., Unger T., Dym O., Prilusky J., Sussman J.L., Stevens R.C., Lesley S.A., Wilson I.A., Joachimiak A., Collart F., Dementieva I., Donnelly M.I., Eschenfeldt W.H., Kim Y., Stols L., Wu R., Zhou M., Burley S.K., Emtage J.S., Sauder J.M., Thompson D., Bain K., Luz J., Gheyi T., Zhang F., Atwell S., Almo S.C., Bonanno J.B., Fiser A., Swaminathan S., Studier F.W., Chance M.R., Sali A., Acton T.B., Xiao R., Zhao L., Ma L.C., Hunt J.F., Tong L., Cunningham K., Inouye M., Anderson S., Janjua H., Shastry R., Ho C.K., Wang D., Wang H., Jiang M., Montelione G.T., Stuart D.I., Owens R.J., Daenke S., Schutz A., Heinemann U., Yokoyama S., Bussow K., Gunsalus K.C. (2008). Protein production and purification. Nat. Methods.

[bib17] Hart D.J., Tarendeau F. (2006). Combinatorial library approaches for improving soluble protein expression in *Escherichia coli*. Acta Crystallogr. D Biol. Crystallogr..

[bib18] Hedhammar M., Hober S. (2007). Z(basic)–a novel purification tag for efficient protein recovery. J. Chromatogr. A.

[bib19] Jones D.T. (1999). Protein secondary structure prediction based on position-specific scoring matrices. J. Mol. Biol..

[bib20] Lennon G., Auffray C., Polymeropoulos M., Soares M.B. (1996). The I.M.A.G.E. Consortium: an integrated molecular analysis of genomes and their expression. Genomics.

[bib21] Linding R., Russell R.B., Neduva V., Gibson T.J. (2003). GlobPlot: exploring protein sequences for globularity and disorder. Nucleic Acids Res..

[bib22] Luria S.E., Delbruck M. (1943). Mutations of Bacteria from Virus Sensitivity to Virus Resistance. Genetics.

[bib23] Magnusdottir A., Johansson I., Dahlgren L.G., Nordlund P., Berglund H. (2009). Enabling IMAC purification of low abundance recombinant proteins from *E. coli* lysates. Nat. Methods.

[bib24] Marchler-Bauer A., Bryant S.H. (2004). CD-Search: protein domain annotations on the fly. Nucleic Acids Res..

[bib25] McGuffin L.J., Jones D.T. (2003). Improvement of the GenTHREADER method for genomic fold recognition. Bioinformatics.

[bib26] McGuffin L.J., Bryson K., Jones D.T. (2000). The PSIPRED protein structure prediction server. Bioinformatics.

[bib27] Page R., Moy K., Sims E.C., Velasquez J., McManus B., Grittini C., Clayton T.L., Stevens R.C. (2004). Scalable high-throughput micro-expression device for recombinant proteins. BioTechniques.

[bib28] Pearl L.H. (2005). Hsp90 and Cdc37–a chaperone cancer conspiracy. Curr. Opin. Genet. Dev..

[bib29] Prilusky J., Felder C.E., Zeev-Ben-Mordehai T., Rydberg E.H., Man O., Beckmann J.S., Silman I., Sussman J.L. (2005). FoldIndex: a simple tool to predict whether a given protein sequence is intrinsically unfolded. Bioinformatics.

[bib30] Pruitt K.D., Tatusova T., Maglott D.R. (2007). NCBI reference sequences (RefSeq): a curated non-redundant sequence database of genomes, transcripts and proteins. Nucleic Acids Res..

[bib31] Reich S., Puckey L.H., Cheetham C.L., Harris R., Ali A.A., Bhattacharyya U., Maclagan K., Powell K.A., Prodromou C., Pearl L.H., Driscoll P.C., Savva R. (2006). Combinatorial domain hunting: an effective approach for the identification of soluble protein domains adaptable to high-throughput applications. Protein Sci..

[bib32] Savchenko A., Yee A., Khachatryan A., Skarina T., Evdokimova E., Pavlova M., Semesi A., Northey J., Beasley S., Lan N., Das R., Gerstein M., Arrowsmith C.H., Edwards A.M. (2003). Strategies for structural proteomics of prokaryotes: quantifying the advantages of studying orthologous proteins and of using both NMR and X-ray crystallography approaches. Proteins.

[bib33] Schultz J., Milpetz F., Bork P., Ponting C.P. (1998). SMART, a simple modular architecture research tool: identification of signaling domains. Proc. Natl. Acad. Sci. USA.

[bib34] Shrestha B., Smee C., Gileadi O. (2008). Baculovirus expression vector system: an emerging host for high-throughput eukaryotic protein expression. Methods Mol. Biol..

[bib35] Slabinski L., Jaroszewski L., Rychlewski L., Wilson I.A., Lesley S.A., Godzik A. (2007). XtalPred: a web server for prediction of protein crystallizability. Bioinformatics.

[bib36] Stols L., Gu M., Dieckman L., Raffen R., Collart F.R., Donnelly M.I. (2002). A new vector for high-throughput, ligation-independent cloning encoding a tobacco etch virus protease cleavage site. Protein Expr. Purif..

[bib37] Temple G., Gerhard D.S., Rasooly R., Feingold E.A., Good P.J., Robinson C., Mandich A., Derge J.G., Lewis J., Shoaf D., Collins F.S., Jang W., Wagner L., Shenmen C.M., Misquitta L., Schaefer C.F., Buetow K.H., Bonner T.I., Yankie L., Ward M., Phan L., Astashyn A., Brown G., Farrell C., Hart J., Landrum M., Maidak B.L., Murphy M., Murphy T., Rajput B., Riddick L., Webb D., Weber J., Wu W., Pruitt K.D., Maglott D., Siepel A., Brejova B., Diekhans M., Harte R., Baertsch R., Kent J., Haussler D., Brent M., Langton L., Comstock C.L., Stevens M., Wei C., van Baren M.J., Salehi-Ashtiani K., Murray R.R., Ghamsari L., Mello E., Lin C., Pennacchio C., Schreiber K., Shapiro N., Marsh A., Pardes E., Moore T., Lebeau A., Muratet M., Simmons B., Kloske D., Sieja S., Hudson J., Sethupathy P., Brownstein M., Bhat N., Lazar J., Jacob H., Gruber C.E., Smith M.R., McPherson J., Garcia A.M., Gunaratne P.H., Wu J., Muzny D., Gibbs R.A., Young A.C., Bouffard G.G., Blakesley R.W., Mullikin J., Green E.D., Dickson M.C., Rodriguez A.C., Grimwood J., Schmutz J., Myers R.M., Hirst M., Zeng T., Tse K., Moksa M., Deng M., Ma K., Mah D., Pang J., Taylor G., Chuah E., Deng A., Fichter K., Go A., Lee S., Wang J., Griffith M., Morin R., Moore R.A., Mayo M., Munro S., Wagner S., Jones S.J., Holt R.A., Marra M.A., Lu S., Yang S., Hartigan J., Graf M., Wagner R., Letovksy S., Pulido J.C., Robison K., Esposito D., Hartley J., Wall V.E., Hopkins R.F., Ohara O., Wiemann S. (2009). The completion of the Mammalian Gene Collection (MGC). Genome Res..

[bib38] Uhlen M., Graslund S., Sundstrom M. (2008). A pilot project to generate affinity reagents to human proteins. Nat. Methods.

[bib39] Vedadi M., Niesen F.H., Allali-Hassani A., Fedorov O.Y., Finerty P.J., Wasney G.A., Yeung R., Arrowsmith C., Ball L.J., Berglund H., Hui R., Marsden B.D., Nordlund P., Sundstrom M., Weigelt J., Edwards A.M. (2006). Chemical screening methods to identify ligands that promote protein stability, protein crystallization, and structure determination. Proc. Natl. Acad. Sci. USA.

[bib40] Vincentelli R., Bignon C., Gruez A., Canaan S., Sulzenbacher G., Tegoni M., Campanacci V., Cambillau C. (2003). Medium-scale structural genomics: strategies for protein expression and crystallization. Acc. Chem. Res..

[bib41] Williamson A.R. (2000). Creating a structural genomics consortium. Nat. Struct. Biol..

[bib42] Yancey P.H. (2005). Organic osmolytes as compatible, metabolic and counteracting cytoprotectants in high osmolarity and other stresses. J. Exp. Biol..

