# Supplementary material for: High-throughput production of human proteins for crystallization: The SGC experience
Source: J Struct Biol. 2010 Oct;172(1):3–13. doi: 10.1016/j.jsb.2010.06.008 (PMC2938586; doi:10.1016/j.jsb.2010.06.008)
Supplement: Supplementary data 1 — Vector information sheet. [file mmc1.pdf]

Vector information sheet.

Vector Name	**pNIC28-Bsa4**
Source	Opher Gileadi
Sequence accession/link	Genebank EF198106

Description	pET expression vector with His_6 tag in 22-aa N-terminal fusion peptide, with TEV protease cleavage site. Includes sites for LIC cloning, and a “stuffer” fragment that includes the SacB gene, allowing negative selection on 5% sucrose

Antibiotic resistance	Kanamycin, 50 µg/ml
Promoter	T7 - lacO
Cloning	LIC. (vector treated with BsaI, then with T4 DNA polymerase in presence of dGTP)
Initiation codon	Supplied in PCR primer
N-terminal fusion – seq.	MHHHHHHSSGVDLGTENLYFQ*SM<br>(* - TEV cleavage site)
N-terminal fusion – MW	2684.1 Da including Met (2465.8 Da removed by TEV cleavage)
Termination codons	supplied in PCR primer
Protease cleavage	TEV
Additional features	
Preferred host	DE3 hosts: BL21, Rosetta, etc. MUST express T7 RNA polymerase.
5’ sequencing primer	pLIC-for: TGTGAGCGGATAACAATTCC
3’ sequencing primer	pLIC-rev: AGCAGCCAACTCAGCTTCC

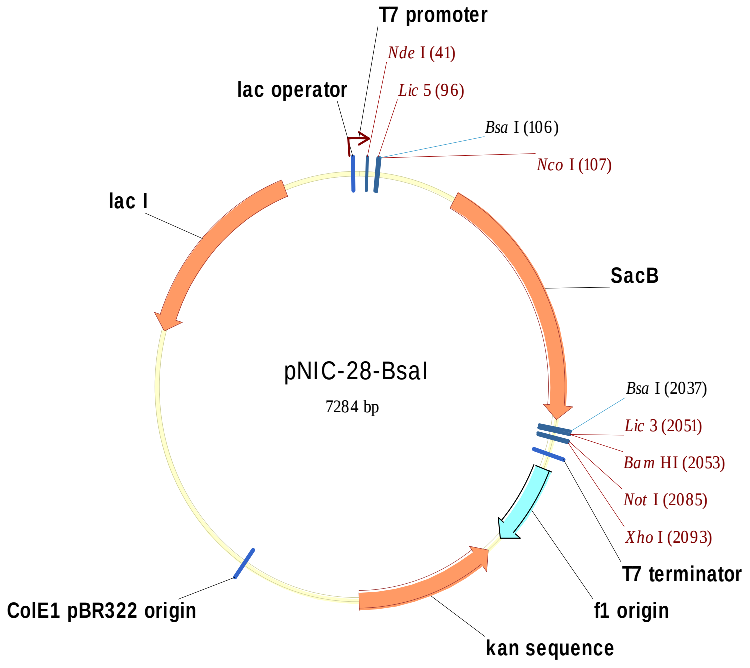

Opher Gileadi, Structural Genomics Consortium
opher.gileadi@sgc.ox.ac.uk

Polylinker region

```
                               T7-forward                pLIC-forward
                          -------------------->       -------------------->
                                                       lac operator
                                                ~~~~~~~~~~~~~~~~~~~~~~~~~~~
7222    CTCGATCCCG CGAAATTAAT ACGACTCACT ATAGGGGAAT TGTGAGCGGA TAACAATTCC
        GAGCTAGGGC GCTTTAATTA TGCTGAGTGA TATCCCCTTA ACACTCGCCT ATTGTTAAGG

                                                         NdeI
                                                        ~~~~~~
                                                            M  H   H  H  H  H ·
7282    CCTCTAGAAA TAATTTTGTT TAACTTTAAG AAGGAGATAT ACATATGCAC CATCATCATC
        GGAGATCTTT ATTAAAACAA ATTGAAATTC TTCCTCTATA TGTATACGTG GTAGTAGTAG

                                                      Upper-LIC        BsaI
                                                   ~~~~~~~~~~~~~~~~  ______
        · H  S  S   G  V  D   L  G  T  E   N  L  Y   F  Q  S
58      ATCATTCTTC TGGTGTAGAT CTGGGTACCG AGAACCTGTA CTTCCAATCC ATGGAGACCG
        TAGTAAGAAG ACCACATCTA GACCCATGGC TCTTGGACAT GAAGGTTAGG TACCTCTGGC

118     ACGTCCACAT .........    (SacB fragment)   .....................
        TGCAGGTGTA

                                                                  EcoRI
                             BsaI      Lower-LIC        BamHI ~~~~~~~ SacI
                            ______  ~~~~~~~~~~~~~~~~ ~~~~~~        ~~~~~~
2010    GATATCCTAT TGGCATTGAC GGTCTCCAGT AAAGGTGGAT ACGGATCCGA ATTCGAGCTC
        CTATAGGATA ACCGTAACTG CCAGAGGTCA TTTCCACCTA TGCCTAGGCT TAAGCTCGAG

          SalI
               HindIII
         ******~~~~~~~
2070    CGTCGACAAG CTTGCGGCCG CACTCGAGCA CCACCACCAC CACCACTGAG ATCCGGCTGC
        GCAGCTGTTC GAACGCCGGC GTGAGCTCGT GGTGGTGGTG GTGGTGACTC TAGGCCGACG
                                                        T7-reverse
                                                    <-----------------
2130    TAACAAAGCC CGAAAGGAAG CTGAGTTGGC TGCTGCCACC GCTGAGCAAT AACTAGCATA
        ATTGTTTCGG GCTTTCCTTC GACTCAACCG ACGACGGTGG CGACTCGTTA TTGATCGTAT
                         <-------------------
                              pLIC-rev
```

Primers for LIC cloning:

Upstream: add TACTTCCAATCC<u>ATG</u> to the 5’ end (ATG in-frame with the desired coding sequence).

Downstream: add TATCCACCTTTACTG to 5’ end of downstream primer; add termination codon, if necessary.

Opher Gileadi, Structural Genomics Consortium
opher.gileadi@sgc.ox.ac.uk
