# Supplementary material for: High-throughput production of human proteins for crystallization: The SGC experience
Source: J Struct Biol. 2010 Oct;172(1):3–13. doi: 10.1016/j.jsb.2010.06.008 (PMC2938586; doi:10.1016/j.jsb.2010.06.008)
Supplement: Supplementary data 2 — Experimental details for LIC cloning vectors (see Table 1 in the text for full vector details). [file mmc2.doc]

| **Vector** | **Antibiotic Resistance** | **Digest Buffer** | **Digest Enzyme** | **Digest Conditions** | **dNTP for T4 pol treatment of VECTOR** | **dNTP for T4 pol treatment of INSERT** | **5’ extensions on PCR primers** | **Colony PCR Primers** |
| --- | --- | --- | --- | --- | --- | --- | --- | --- |
| pLIC-SGC1 | Amp | NEB2 | BseRI | 37^o^C, 2-3 h, add more enzyme | dGTP | dCTP | 1 | pLIC for / pLIC rev |
| pFB-LIC-Bse | Amp | NEB2 | BseRI | 37^o^C, 2-3 h, add more enzyme | dGTP | dCTP | 1 | Fbac-1 / Fbac-2 |
| pNIC28-Bsa4 | Kan | NEB3 | BsaI | 50^o^C, 2-3 h | dGTP | dCTP | 1 | pLIC for / pLIC rev |
| pNH-TrxT | Kan | NEB3 | BsaI | 50^o^C, 2-3 h | dGTP | dCTP | 1 | pLIC for / pLIC rev |
| pNIC-Zb | Kan | NEB3 | BsaI | 50^o^C, 2-3 h | dGTP | dCTP | 1 | pLIC for / pLIC rev |
| pNIC-CTHF | Kan | NEB3 | BfuAI | 50^o^C, O/N | dCTP | dGTP | 2 | pLIC for / pLIC rev |
| pNIC-CH | Kan | NEB3 | BfuAI | 50^o^C, O/N | dCTP | dGTP | 3 | pLIC for / pLIC rev |

Table S1: Experimental details for LIC cloning vectors (see table 1 in the text for full vector details)
