# Supplementary material for: High-throughput production of human proteins for crystallization: The SGC experience
Source: J Struct Biol. 2010 Oct;172(1):3–13. doi: 10.1016/j.jsb.2010.06.008 (PMC2938586; doi:10.1016/j.jsb.2010.06.008)
Supplement: Supplementary data 4 [file mmc4.doc]

**Supplementary material.**

**Contents:**

1. Detailed materials and methods.
2. Figure S1: diversity and difficulty of SGC targets.
3. Table S2 – cloning vectors
4. Table S3 – Structures solved by SGC: details of construct sequence and protein purification schemes.
5. Figure S4 – details of plasmid pNIC28-Bsa4.

**Materials and Methods.**

The following protocols describe high-throughput cloning and expression in a 96 well format (a step by step protocol can be found in [1]). If low throughput is required these protocols can be scaled down accordingly.

1. Buffers and reagents

1.1 Affinity buffer: 50 mM HEPES-NaOH, pH 7.5, 0.5 M NaCl, 10 mM imidazole, 1 mM TCEP.

*Comment: 50 mM Na-phosphate buffer can be used instead of HEPES in buffers 1.1 – 1.6; the lysis buffer can also be supplemented with 5-10 % glycerol.*

1.2 Bugbuster buffer: 50 mM HEPES, pH 7.5, 0.5 M NaCl, 10 mM imidazole, 1 mM TCEP, 1/10 vol of Bugbuster® 10x concentrate (Novagen), 25 units/ml benzonase (Novagen), and 1x protease inhibitor cocktail (Calbiochem cocktail IV).

1.3 Lysis buffer: Affinity buffer supplemented with 1x protease inhibitors.

1.4 2x lysis buffer: Lysis buffer at 2x concentration

1.5 Wash buffer: 50 mM HEPES, pH 7.5, 0.5 M NaCl, 30 mM imidazole, 1 mM TCEP

1.6 Elution buffer: 50 mM HEPES, pH 7.5, 0.5 M NaCl, 300 mM imidazole, 1 mM TCEP

1.7 Gel filtration buffer: 10 mM HEPES, pH 7.5, 0.5 M NaCl, 5% glycerol, 1 mM TCEP.

1.8 PEI: 5% polyethylenimine (10x dilution of original 50% stock, Sigma), adjusted to pH 7.5 with HCl.

Benzonase (HC) and Bugbuster (10x) are from Novagen (Merck); NiNTA-agarose beads are from Qiagen, equilibrated in affinity buffer before use. Restriction enzymes and T4 DNA polymerase are from New England Biolabs (NEB).

1.9 Oligonucleotides: PCR primers for LIC cloning depend on the vector. All primers bear extensions on the 5’ end that overlap vector sequences flanking the cloning site.

Group 1: (pNIC28-Bsa4, pFB-LIC-Bse etc., see table S2)

Upstream: add TACTTCCAATCCatg to the 5’ end (atg is in-frame with the desired coding sequence).

Downstream: add TATCCACCTTTACTGtca to 5’ end of downstream primer (the lowercase tca is the complement of a termination codon).

Group 2: (pNIC-CTHF):

Upstream: Add TTAAGAAGGAGATATACTatg (atg-initiation codon)

Downstream: GATTGGAAGTAGAGGTTCTCTGC (no termination codon!)

Group 3:

Upstream: TTAAGAAGGAGATATACTatg (atg-initiation codon)

Downstream: AATGGTGGTGATGATGGTGCGC

Primers for colony PCR and sequencing:

All bacterial vectors:

pLIC-for: TGTGAGCGGATAACAATTCC

pLIC-rev: AGCAGCCAACTCAGCTTCC

Baculovirus transfer vectors:

FBAC1: TATTCATACCGTCCCACCA

FBAC2: GGGAGGTTTTTTAAAGCAAGTAAA

2. Cloning

*2.1 96-Well PCR.*

To simplify the assembly of 96 PCR reactions and to allow easy repetition or optimization, it is useful to prepare separate master plates of templates and primers. The forward and reverse primers for each reaction are combined in wells of a 96-well plate and diluted with PCR-grade water to a concentration of 2.5 μM each. The templates are diluted to 1 ng/μl and aliquoted into the corresponding wells of a separate 96-well plate. In our experience, Pfx Platinum DNA polymerase (invitrogen) works reasonably well in most cases and we use it as standard. In some rare cases when Pfx works poorly it can be substituted with Herculase (Stratagene) or Taq DNA polymerase. PCR reactions are set up in a fresh 96-well plate, using a master mix containing (for 100 reactions): 1350 μl of water, 500 μl of 10x Pfx amplification buffer, 50 μl of 50 mM MgSO_4_, 75 μl of dNTP mix (10 mM each) and 25 μl of Pfx enzyme. Twenty μl are dispensed into each well. Then, 2.5 μl of the primer mix and 2.5 μl of diluted template are added to each well using a multichannel pipettor and the plate is sealed with a heat-resistant adhesive film (Abgene).

We found that “touchdown PCR” generally produces better results than a uniform cycle protocol. The following thermocycler program is routinely used: 95°C, 5 min for initial template denaturation/hot start, followed by 5 cycles of 95°C, 30 seconds, 68°C, x minutes; 5 cycles of 95°C, 30 seconds, 60°C, 30 seconds, 68°C, x minutes; 5 cycles of 95°C, 30 seconds, 55°C, 30 seconds, 68°C, x minutes; and 20 cycles of 95°C, 30 seconds, 50°C, 30 seconds, 68°C, x minutes followed by final extension at 68°C for 5 minutes. The length of the extension step (x) depends on the expected fragment size; allow approximately 1 min/kb plus 30 seconds. If the template plasmid and the cloning vector have the same antibiotic resistance, the remaining template DNA is digested with the methyl-specific restriction enzyme DpnI: add 2.5 µl of 10xNEBuffer 2 (New England Biolabs) and 2 units DpnI, incubate for 30 minutes at 37°C. The PCR products are purified using a 96-well PCR purification kit (e.g. from Millipore or Qiagen).

*2.2 Cohesive End Generation (Vector).*

Five μg of the cloning vector are cut with the appropriate restriction enzyme (Table S2). The recommended digestion conditions are:

BsaI: 100 ul reaction containing 10 µl 10xNEBuffer 3, 1 µl BSA (10 mg/ml), water to 97 µl, and 3 µl BsaI (30 units). Incubate 2 – 3 hours at 50°C

BseRI: 100 ul reaction containing 10 µl 10xNEBuffer 2, 1 µl BSA (10 mg/ml), 5 µg DNA, water to 94 µl, and 6,25 µl BseRI (25 units). After 2 hours at 37°C, add 20 µl water, 2.5 µl NEBuffer 2, and 2.5 µl BseRI; incubate for another hour. Overdigestion with this enzyme may lead to non-specific cleavage.

BfuAI: 200 µl reaction containing 20 µl 10xNEBuffer 3, 5 µg DNA, water to 180 µl, and 20 µl BfuAI (80 units). Incubate overnight at 50°C.

The DNA is purified using a PCR purification column (Qiagen) and eluted in 50 μl EB (10 mM tris-HCl, pH 8.5). To generate cohesive ends, the digested vector is treated with T4 DNA polymerase in the presence of appropriate nucleotide. Mix 15 μl water, 50 μl *Bsa*I-digested plasmid, 10 μl 10x NEB2 buffer (New England Biolabs), 10 μl of 25 mM deoxynucleotide (Table S2), 1 μl BSA (10 mg/ml, NEB), 5 μl 100mM DTT, 5 μl T4 DNA polymerase (NEB) and incubate 30 minutes at 22°C, then inactivate at 80°C for 30 min.

*2.3 Cohesive End Generation (Inserts)*

The master mix (100 reactions) contains100 μl of 10xNEBuffer 2, 100 μl of 25 mM deoxynucleotide (Table S2), 240 μl of water, 50 μl of 100 mM DTT, 10 μl of BSA (NEB, 10 mg/ml) and 5 μl of T4 DNA polymerase (NEB). Five μl are dispensed into each well of a 96-well PCR plate. Five μl of each purified PCR fragment is added to the corresponding well with a multichannel pipette and mixed by pipetting gently up and down once. The plate is sealed and incubated for 30 minutes at 22°C, then 30 minutes at 80°C to inactivate the enzyme.

*2.4 Annealing and Transformation*

One μl of the treated vector and 2 μl of treated inserts are mixed in a fresh 96-well PCR plate. Competent *E.coli* cells (25 μl of Mach1 or other T1 phage-resistant strain) are added to each well; after a 15-min incubation on ice and a 30 sec heat shock at 42°C, 100 μl of SOC medium are added and the plate is incubated at 37°C for 60 minutes. Each transformation mixture is plated on a 60-mm petri dish containing LB-agar with 5% sucrose and the appropriate antibiotic (50 μg/ml kanamycin or 200 μg/ml ampicillin). The plates are incubated at 37°C overnight until colonies appear.

*2.5 Colony Screening.*

Individual colonies are screened by PCR using vector-specific primers. As the PCR products are not used for cloning, a non-proofreading polymerase (e.g. Biotaq Red DNA polymerase) can be used. A master mix for 100 reactions contains 1600 µl water, 200 µl 10xbuffer, 60 µl DMSO, 60 µl of 50 mM MgCl_2_, 40 µl of 10 mM dNTP mix, 10 µl each primer, and 40 µl enzyme (prepared on ice). 20 µl of the master mix is dispensed in each well of a 96-well PCR plate. A single colony is scraped with a disposable inoculation loop, which is swirled in the PCR mix, then transferred to 1ml of medium LB/antibiotic in a deep-well block. The deep-well block is sealed and allowed to grow overnight for glycerol stocks and to inoculate miniprep cultures. The PCR plates are sealed and transferred to a pre-heated thermocycler programmed as above. The PCR reactions are analyzed on a agarose-TAE gel and the fragments scrutinized carefully to identify bands of the correct size. Additional colonies may be tested if the first colony does not yield the correct fragment.

*2.6 Expression clones*

The plasmids are purified from the selected colonies using a 96-well miniprep kit (Millipore or Qiagen), according to the supplier’s instructions. The plasmid yields are typically 30-50 ng/µl or 1.5-2.5 µg in total.

Five µl of each purified plasmid is used to transform the expression host BL21(DE3)-R3-pRARE; the transformation mix is plated onto LB -agar containing the relevant antibiotic plus chloramphenicol (to maintain the rare-codon tRNA plasmids pRARE2). For expression, several colonies are combined into 1 ml of medium, grown for several hours or overnight, supplemented with glycerol to 15% (v/v) and stored at -80°C.

3. Small-Scale Test Expression.

*3.1 Cell Growth and Induction.*

Starter cultures (1 ml) in TB+antibiotics are initiated either from the original colonies or by inoculating each well of a 96 deep-well block with a lump of cells from the glycerol stocks. The block is covered with a porous seal and the cultures grown overnight with vigorous shaking at 37°C in a Glas-col shaker at 700 RPM. Next day, a fresh 96 deep-well block is inoculated with 20 μl of overnight cultures and grown at 37°C until OD_600_ reaches 2. The temperature is lowered to 18 – 25°C for 30 minutes, then 10 μl of 10 mM IPTG is added. Incubation is continued overnight at the same temperature. The cells are collected by centrifugation (4000 x g, 15 minutes), the supernatants are poured off and destroyed. The block may be stored at −80°C or processed directly.

*3.2 Small-Scale Purification and Analysis.*

The cells are thawed completely (if frozen), and vortexed to disperse the pellets completely. Bugbuster buffer (400 μl ) is added to each well vortexed thoroughly. The block is incubated at 4°C for 30 minutes with occasional shaking to degrade cellular DNA. Aliquots (20 μl) are saved from each well for later analysis as “total extract”; the block is centrifuged at 4000 RPM for 20 min, and the supernatants are transferred to a new block using a multichannel pipettor. NiNTA beads (50 μl of a 50 % slurry) are added to each well and the block is incubated for 1 hour at 4°C with 400 rpm shaking. A 96-well filter block is placed over a new 96 deep-well block (drain block) and the lysates with the beads are transferred into the filter plate. The filter and drain blocks are then centrifuged for 1 minute at 500xg, and discard the flow-through. The beads in the filter block are washed 3 times by adding 800 μl of wash buffer to each well, and then centrifuging as above. The filter block is then placed on top of a microtiter plate, elution buffer (50 μl) is added directly onto the beads and incubated for 5 minutes. The eluted proteins are collected by centrifugation for 2 minutes at 500xg. The total and eluted fractions are analyzed by SDS-PAGE and coomassie staining.

4. Standard Scale-up Protocol.

*4.1 Culture Growth and Induction.*

Starter cultures are inoculated from glycerol stocks or with fresh colonies and grown overnight at 37°C in a shaker. Ten ml of the overnight culture is used to inoculate 1L of TB medium in a baffled flask (Ultrayield, Thomson), which is then incubated at 37°C with 200 rpm shaking. When the OD_600_ reaches 2.00 ± 1, the temperature is lowered to 18°C and incubation continued for another hour until the medium cools down. IPTG is added (0.1 mM), and incubation is continued overnight at 18°C. The cells are harvested by centrifugation, the supernatant is safely disposed, and the cells are scraped and weighed. The cell pellet is resuspended in 1 ml/gr of 2x Lysis Buffer and freozen at -80°C or processed directly.

*4.2 Cell Extraction.*

The cell pellets are thawed at room temperature and then transferred to ice. Protease inhibitors are added and the cells are completely resuspended by extensive vortexing. The cells are lysed in a high-pressure cell homogenizer according to the instrument instructions. The homogenizer is flushed with lysis buffer until the effluent is clear (20–40 ml) and combine with the lysate. Alternatively, cells are lysed using sonication. Polyethyleneimine (PEI) is added to a final concentration of 0.15%, the lysate is incubated for 15 minutes on ice and centrifuged at 17,000 rpm for 30 minutes at 4°C. The clarified lysate is decanted to a clean beaker and the insoluble pellets of insoluble proteins, nucleic acids and cell debris are discarded.

*4.3 Affinity Protein Purification.*

Affinity purification can be done in an automated fashion using AKTA-express system according to manufacturer’s protocol or manually using gravity flow columns.

*4.3.1 Manual Affinity Purification.*

NiNTA resin equilibrated in in affinity buffer is added to the cell lysate in conical 50-ml tubes (use 1-5 ml of 50 % slurry per liter of culture, depending on the estimated expression level). The tubes are mixed by slow tumbling for at least 1 hour at 4°C and then transferred to a column (alternatively, the protein can be bound to the resin in the column by gravity flow). The resin is washed with 10 column volumes of affinity buffer and then with 20 column volumes of wash buffer. The protein is eluted with 10 column volumes of elution buffer, collecting fractions of 0.5 column volumes. Aliquots are analyzed by SDS-PAGE for presence and purity of the recombinant protein.

*4.3.2 Automated affinity purification*

Lysates of 1-2L cultures can be purified on 1-ml or 5-ml Histrap columns (GE Healthcare); larger lysates should be purified on 5-ml columns at a high flow rate (5 ml/min), which prevents leaching of the Ni^2+^ from the columns. The buffer composition and volumes are identical to the manual purification protocol.

*4.3.3 Gel filtration*

Select the gel filtration column according to the expected native size of the protein: Superdex S75 or S200, HR 16/60 columns are used at 1.2 ml/min in GF buffer. Protein from manual NiNTA purification can be concentrated prior to injection; when using automated purification on AKTA-express, the Histrap eluate is collected automatically and re-injected onto the gel filtration column. Fractions of 1.8 ml are collected and aliquots are analyzed by SDS-PAGE.

*4.3.4 Further purification.*

Tag cleavage.

The protein is digested with TEV protease (1:20 mol/mol ratio) overnight at 4°C to remove the tag. The protease is fused to a His10 sequence, and can be removed alongside the cleaved tag and non-specific Ni-binding proteins by passing through a NiNTA column (1-5 ml). For this protocol to work, the protein solution must contain no more than 30 – 40 mM imidazole; if the protein solution contains imidazole (e.g. if the NiNTA eluate is digested directly), the imidazole should be removed by gel filtration, buffer exchange or dialysis before re-purification.

**Reference**

[1] O. Gileadi, N.A. Burgess-Brown, S.M. Colebrook, G. Berridge, P. Savitsky, C.E. Smee, P. Loppnau, C. Johansson, E. Salah, and N.H. Pantic, High throughput production of recombinant human proteins for crystallography, Methods Mol Biol, **426** (2008) 221-46.
